# Physical Activity and Fall Prevention in Geriatric Inpatients in an Acute Care Unit (AGIR Study): Protocol for a Usability Study

**DOI:** 10.2196/32288

**Published:** 2022-07-11

**Authors:** Frédéric Noublanche, Romain Simon, Grégory Ben-Sadoun, Cédric Annweiler

**Affiliations:** 1 Department of Geriatric Medicine and Memory Clinic Research Center on Autonomy and Longevity University Hospital of Angers Angers France; 2 Laboratoire de Psychologie des Pays de la Loire Université Angers, Université de Nantes EA 4638 LPPL, SFR Confluences, F-49000 Angers France; 3 Normandie Université, UNICAEN, INSERM, COMETE, CYCERON, CHU Caen 14000 Caen France; 4 Robarts Research Institute, Department of Medical Biophysics Schulich School of Medicine and Dentistry The University of Western Ontario London, ON Canada

**Keywords:** fall prevention, physical activity, older patients, geriatric acute care unit

## Abstract

**Background:**

Falls are one of the world’s top 10 risks associated with disability in people older than 60 years. They also represent more than two-thirds of adverse events in hospitals, mainly affecting patients older than 65 years. Physical activity is a central intervention in fall prevention for older people. Whatever the details of the prevention strategy that is adopted (ie, how a mono- or multifactorial intervention is evaluated, the category of person the intervention targets, and where it is used), it is important to ensure that the proposed intervention is feasible and usable for the patient and the health care team.

**Objective:**

The primary objective is to study the usability of carrying out a physical activity intervention, including 3 types of exercises, in older patients hospitalized in a geriatric acute care unit and categorized according to 3 fall risk levels: low, moderate, and high. The secondary objectives are to determine the difficulty of the physical exercise for patients with different fall risk levels, to study the health care team’s perceptions of the intervention’s feasibility, and to study the benefits for patients.

**Methods:**

This is an open-label, unicenter, nonrandomized, usability prospective clinical trial. The intervention tested is a daily physical activity program. It consists of 3 types of physical exercise: staying out of bed for at least 3 hours, performing balance exercises while standing for 2 minutes, and the Five Times Sit to Stand transfer exercise. These exercises are carried out under the supervision of the health care team. Fall risk in the patients is classified with the Brief Geriatric Assessment tool. The exercise program starts on the second day of hospitalization after inclusion in the study. Patient assessment continues until the last day of hospitalization or the 20th day of hospitalization, whichever is earlier. For each fall-risk group and each type of exercise, the intervention will be defined as usable if at least 80% of the participants complete 75% or more of the exercises (ie, the ratio between the number of days when the patient completes a type of exercise and the total number of hospitalization days). The perceived feasibility by the health care team is measured with 2 scales, measuring perceived difficulty and time spent with the patient. The intervention benefit is evaluated using the performance of the Five Times Sit to Stand test before and after the intervention.

**Results:**

The first patient was recruited on March 16, 2015. The study enrolled 266 patients, including 75 with low fall risk, 105 with moderate risk, and 85 with high risk.

**Conclusions:**

We have not yet analyzed the results, but our observations suggest that the usability of each type of exercise for a given patient will depend on their fall risk level.

**Trial Registration:**

ClinicalTrials.gov NCT02393014; https://clinicaltrials.gov/ct2/show/NCT02393014

**International Registered Report Identifier (IRRID):**

DERR1-10.2196/32288

## Introduction

### Context

Falls are one of the world’s top 10 risks for causing disability in people aged older than 60 years [1]. More than two-thirds of adverse events in hospitals are falls, mainly affecting patients older than 65 years [2,3]. Falls are accompanied by multiple physical and psychological consequences that cause disability, increased length of stay, and increased cost of care [2,3]. Fall risk factors may be classified as (1) biological (related to disease or aging), (2) associated with daily behavior (related to eating, being active, dressing, and other habits), (3) social and economic (related to social isolation, poverty, financial resources, or lack of access to health care), and (4) environmental, including the local environment (building entrances, lack of handrails, type of furniture) and the climate [4]. The management of falls relies on various strategies: avoiding the first fall, fast intervention and treatment when a fall occurs, and preventing additional falls [5].

Physical activity is a central intervention in fall prevention programs for older persons. It contributes to the restoration and maintenance of muscle function and tone, joint mobility, improved balance, and walking ability [5-8]. Physical activity recommendations for older people [9], particularly frail patients at risk of falling [5,6], include moderate-intensity physical activity for 15 to 30 minutes as often as possible during the week; exercise programs based on balance, strength, and gait; group exercise supervised by a professional, to take into account the physical capabilities and health profile of older persons; and regular review of progression to adjust the exercise prescription as appropriate. Recommendations also include multicomponent interventions [10]. It has been shown that multicomponent interventions, which combine physical activity and corrective actions, decrease the annual incidence of falls by 10% to 30% [6,7].

Staying active prevents functional disabilities, particularly in older patients who are hospitalized [11]. These patients quickly lose their ability to perform daily acts by themselves, such as getting up from a low position or walking, causing them to become severely sedentary [6-8,12]. In this context, an intervention can be considered a secondary or tertiary prevention (ie, taking place after confirmation of early-stage risk factors, such as frailty or a history of falls) rather than a primary prevention (ie, before the onset of the disease) [13].

Considering (1) the preventive strategy adopted (ie, the evaluation associated with a mono- or multicomponent intervention), (2) the category of person targeted, and (3) where the intervention takes place (ie, in a home, institution, or hospital), it is important to ensure that the proposed intervention is feasible and usable for the person or the group of people concerned. This is particularly important considering the frailty of older inpatients and the availability of the health care team.

### Hypothesis

We hypothesize that in the geriatric acute care unit of a university hospital, it will be possible to carry out a physical activity intervention based on the physical acts of daily life, focusing on strengthening the lower-limb muscles and improving postural balance.

### Objectives

#### Primary Objective

The primary objective is to study the usability of carrying out a physical activity intervention including 3 types of exercise in older inpatients at a geriatric acute-care unit and categorized according to 3 fall risk levels: low, moderate, and high.

#### Secondary Objectives

There are 3 secondary study objectives. Secondary objective A will be to determine the difficulty level of the physical exercises in each fall risk group and analyze the usability of the intervention in each group to determine the best intervention modality for each group. Secondary objective B will be to determine the health care team’s perception of the difficulty of carrying out the intervention and analyze their perceptions with 2 scales. Secondary objective C will be to examine the effects of this physical activity intervention on the patients’ lower-limb strength, comparing their ability and time to perform the Five Times Sit to Stand (FTSS) test before (measured during a clinical assessment) and after the intervention (on the last day of hospitalization or the 20th day of hospitalization, if the patient is still hospitalized).

## Methods

### Design

**Figure 1:**
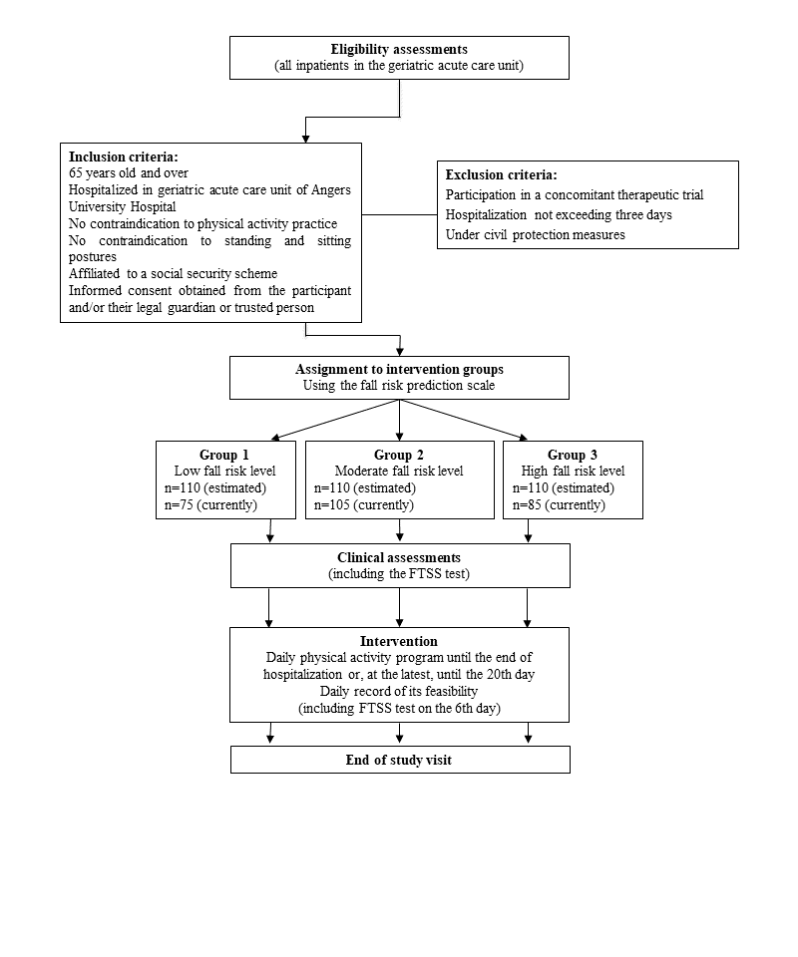
Trial flow chart. FTSS: Five Times Sit to Stand.

This is an open-label, unicenter, nonrandomized, usability prospective clinical trial. There is no comparator group (ie, no control group). Figure 1 illustrates the trial design and Table 1 summarizes the timing of the trial. The intervention tested in this study is a physical activity intervention based on the physical acts of daily life that focuses on strengthening the lower-limb muscles and improving postural balance. It consists of 3 types of physical exercise that can be adapted to the fall risk level. It is offered to 3 categories of hospitalized patients, classified according to their risk of falling: low, moderate, and high. It is carried out from the second day of hospitalization after an assessment of the patient’s risk of falling until the last day of hospitalization or the 20th day of hospitalization if the patient is still hospitalized. In summary, this study consists of an inclusion visit, a physical activity program performed daily until the 20th day of hospitalization, and an end-of-study visit.

**Table 1:** Timeline of the study.

	Enrollment and allocation	Follow up	Study end (last day of hospitalization or 20th day, whichever is earlier)
Inclusion and exclusion criteria applied	✓		
Informed consent obtained	✓		
Group assignment	✓		
Clinical assessment	✓		✓
Five Times Sit to Stand test	✓	✓^a^	✓
Pain	✓	✓^a^	✓
Physical activity intervention		✓^b^	✓

^a^Only measured on the sixth day of follow up.

^b^Performed daily.

### Planned Eligibility Criteria

#### Inclusion Criteria

Patients are included if they are at least 65 years old, hospitalized in a geriatric acute-care unit, and have no contraindications to physical activity, standing, or sitting. Patients must also be covered by a social security scheme and have given and signed an informed consent form to participate in the study (informed consent can also be obtained from a trusted person or legal representative, as appropriate).

#### Exclusion Criteria

Patients are excluded if their hospitalization was shorter than 3 days, they were participating in a concomitant therapeutic trial, or if they were under any of the 3 French civil protection measures: curatorship, guardianship, and safeguard of justice.

### Intervention

This intervention follows the main principles of the latest French National Authority for Health (HAS) recommendations on the prevention of falls in older people [11]. Each fall risk group of participants will receive a physical activity intervention, carried out once a day, consisting of 3 types of exercise. In exercise 1, the participant must stay out of bed for at least 3 hours. This length of time prevents acute postural disorders that increase the risk of falling; these can occur when older patients are bedridden for too long. In exercise 2, the participant stands in static equilibrium for 2 minutes in front of a chair, holding the back of the chair. Depending on the participant’s abilities, the exercise is done standing on one or both feet. Exercise 3 requires the participant to switch from sitting on a chair to standing 5 times (this is similar to the FTSS test but is not timed). Depending on the patient’s abilities, the exercise is done with or without physical assistance.

This daily intervention is carried out starting on the day after informed consent is obtained from patients fulfilling the selection criteria. It is carried out under the supervision and monitoring of the health care team, specifically nurses and nursing assistants.

### Assessments

#### Fall Risk Assessment

The Brief Geriatric Assessment (BGA) tool [14] is used to assess fall risk level. It consists of six items, coded as binary variables: (1) age (over or under 85 years), (2) gender (male or female), (3) number of medications taken daily (over or under 5), (4) history of falls during the past 6 months (yes or no), (5) cognitive level, assessed as the ability to identify the month and year (yes or no), and (6) use of home help services (yes or no). The BGA tool classifies patients into 3 fall risk levels using an algorithm that consider criteria 4 and 5 as major fall risks and criteria 1, 2, 3, and 4 as minor fall risks: group 1, low fall risk; group 2, moderate fall risk; and group 3, high fall risk. The BGA is used by the health care team as part of usual care.

#### Clinical Assessments

Clinical assessments are performed at inclusion and during follow up (Table 1). Except for the FTSS test, which is applied during follow up and at the end of the study, all clinical assessments are carried out as part of usual care. Clinical data are collected from the computerized patient medical file or directly from the participant if the data have not yet been added to the file.

The clinical assessment at inclusion will include the reason for hospitalization (including organ failure, musculoskeletal disorders or falls, neuropsychiatric disorders, medicosocial problems, or other causes); age and gender; anthropometric data (ie, weight and height); body mass index (kg/m^2^); and the Mini-Mental State Examination (MMSE) [15] score. The MMSE is used to assess cognitive function. It consists of 30 questions in 5 sections (orientation, memory and recall, attention, calculation, and language), with a final score graded out of 30 points. In the absence of memory complaint, (self-reported or non–self-reported), a final score between 27 and 30 indicates the probable absence of a cognitive disorder.

Clinical assessments at inclusion and during follow up will include the use of psychotropic drugs (eg, antidepressants, benzodiazepines, hypnotics, and neuroleptics); the number of different therapeutic classes of drugs taken; the FTSS test; and the patient’s overall pain, assessed using a verbal 6-point scale: “no pain,” “low pain,” “moderate pain,” “high pain,” “extreme pain,” and “not assessable.” The pain assessment is used to verify changes in pain level during hospitalization. The FTSS test measures a patient’s physical transfer ability. The test is to stand up from a chair 5 times as quickly as possible without pushing off. Performance is measured in seconds, from the initial seated position to the final seated position after having completed standing up 5 times. A time longer than 15 seconds is abnormal and is associated with physical and cognitive impairment [16].

### Monitoring of the Implementation of the Physical Activity Program

Nurses are in charge of keeping a physical activity monitoring form up to date, which is also digitized in the computerized patient medical file and added to the case report form. This form includes the following information: date of daily physical activity session; level of completion for each type of exercise; reasons why the daily objectives were not reached during the session; perceived feasibility of the daily session by the caregiver; time spent by the caregiver conducting the daily session; and occupational category of the caregiver who supervised the daily session, including nursing assistant, nurse, physiotherapist, occupational therapist, paramedical student, and second-cycle (equivalent to a master’s degree in medical sciences) medical student.

### Outcomes

#### Primary Outcome Measure

The primary outcome measure is the number of participants in each fall risk group who complete 75% or more of the physical activity program for each type of exercise during hospitalization. For each type of exercise, success is quantified daily as follows: for exercise 1, the participant was able to stay out of bed for at least 3 hours; for exercise 2, the participant stood in static balance for at least 2 minutes in front of the chair, holding the back of the chair; and for exercise 3, the participant switched from sitting on a chair to standing at least 5 times. The percentage represents the ratio between the number of days when the patient successfully completes a type of exercise and the total number of days of hospitalization. For each fall risk group and each type of exercise, the intervention will be defined as usable if at least 80% of the participants complete at least 75% of the exercise. These thresholds have not been scientifically validated.

#### Secondary Outcome Measures

The primary outcome will help us to meet secondary objective A. Based on the results for usability, we will establish what types of exercise are usable for each fall-risk group to determine the best intervention modalities for different fall-risk groups in future interventions.

The secondary outcome measures for secondary objective B are the health care team’s perception of the difficulty of carrying out the intervention and the time spent by the health care team on the physical activity intervention. A 5-point scale of perceived difficulty will be assessed after each session, including “very easy,” “easy,” “feasible,” “difficult,” and “impossible.” A scale will also be assessed after each session to quantify the time spent with the patient (ie, the time to complete the physical activity monitoring form, verify the time spent out of bed, and have the patient perform the balance and transfer exercises), including “0 to 2 minutes,” “3 to 5 minutes,” “6 to 10 minutes,” “11 to 20 minutes,” and “>20 minutes.” These scales have not been scientifically validated.

The intervention will be considered feasible if the health care team consider a majority of sessions to have had a difficulty between “very easy” and “feasible” and if a majority of sessions were shorter than 10 minutes.

The secondary outcome measures for secondary objective C are the percentage of patients who completed the FTSS test before and after the intervention and the time in seconds (measured before and after the intervention) the patients needed to complete the FTSS test. We will consider the intervention beneficial for patients’ lower-limb strength in two cases: if the patients can complete the FTSS test more often after the intervention than before, and if those who complete the FTSS test can do so more quickly after the intervention than before.

### Sample Size Calculation

The trial aims to recruit 330 patients, with 110 patients in each of the 3 fall risk groups (Figure 1). It is not possible to accurately calculate the needed size of the population to be studied based on the study’s primary objective. It can be only estimated empirically (creating a risk of error) based on the typology of older patients attending the geriatric acute-care unit involved in this study. The data available to estimate the needed number of participants with this empirical method are the number of hospitalized patients per year (1000 people), the proportion of these patients who cannot complete the proposed exercises due to poor health (approximately 333 people), and the proportion of these patients who can perform the exercises, based on the *autonomie, gérontologie, groupe iso ressources* (AGGIR) a question grid commonly used nation-wide in France [17]. The AGGIR categorizes individuals into *groupe iso ressources* (GIR) levels to represent their level of autonomy. We aimed to include 133, 333, and 200 participants with GIR levels 5 or 6, 3 or 4, and 1 or 2, respectively. We hypothesize that GIR levels will reflect the fall risk levels estimated by the BGA tool.

Considering the proportion of patients who will likely refuse to participate (estimated at 15%), we consider that the study should include at least 330 patients, with the following distribution: 110 patients with low fall risk (GIR levels 5 or 6); 110 patients with moderate fall risk (GIR levels 3 or 4); and 110 patients with major fall risk (GIR levels 1 or 2).

These numbers should be enough to obtain a sufficient proportion of patients who can perform at least 75% of the exercises in each fall risk group and allow us to better understand the usability of the physical activity intervention we are proposing in this study.

### Recruitment/Consent Procedures

The majority of clinical assessments are carried out as part of usual care and as soon as patients enter the geriatric acute-care unit (ie, as soon as possible). This facilitates the preselection of patients, partly thanks to the computerized patient medical file. Once a potential participant is identified as meeting the eligibility criteria, a member of the investigation team provides written and oral information on the study in understandable language to the patient (or a family member, trusted person, or legal representative, as appropriate). When possible, fully informed consent is obtained from the patient. When a patient is unable to give fully informed consent, agreement to participate in the study is obtained from the trusted person or the legal representative, but the patient is not enrolled if he or she refuses or shows significant distress. In the case of exclusion or refusal, the investigation team records whether the selection criteria were met and the cause for nonparticipation in the study, and then copies this information into the “registry of eligible and ineligible patients.”

### Statistical Methods

#### General

The distribution of quantitative variables will be studied, including the mean, median, mode, minimum, maximum, confidence interval around the mean, and standard deviation. The frequencies of qualitative variables will be calculated. For bivariate analyses, the appropriate statistical tests will be used according to the number and distribution of the variables (ie, normal vs nonnormal distributions) and the number of groups of participants in the comparison. The overall significance level will be set at *P=*.05; all tests will be 2-sided.

#### Descriptive Analyses

A diagram will be used to summarize the progress of the study as patients are added and to monitor the protocol. All the clinical characteristics of the participants (such as age, sex, and weight) and the results of the investigations carried out will be described. A table will be used to summarize the level of completion of the physical activity intervention for each fall risk group and each type of exercise. This will help us to determine which type of exercise is most usable for each fall risk group (secondary objective A). A contingency table will be used to report, for each fall risk group, the perceived difficulty of the daily sessions by the health care team and the time spent on carrying them out. A contingency table will also be used to report, for each fall risk group, the number of FTSS tests completed by the patients at the beginning of the intervention, the sixth day of the intervention, and at the end of the intervention.

#### Primary Objective Analysis

For each fall risk group and each type of exercise, the intervention will be considered usable if at least 80% of the participants complete 75% or more of the exercise.

For each parameter, a univariate analysis will be carried out, possibly supplemented by a multivariate analysis, to estimate the effect of physical activity after adjusting for age, sex, and the reason for hospitalization.

#### Comparison Between Fall Risk Groups

The fall risk groups will be compared for (1) the health care team’s perception of their difficulty in carrying out the intervention (secondary objective B) and (2) their results for the FTSS test before and after the intervention (secondary objective C, assessed only in patients who can complete the FTSS test), using parametric and nonparametric tests depending on the distribution of variables.

### Safety Parameters

Safety parameters will be set after adverse events are recorded. The record will include the date of the adverse event; its level of seriousness; the type of effects associated with it (and whether they are known, serious, or unexpected); its novelty (ie, whether is a confounding effect or a new effect previously unknown to us); what it is related to (ranging from unrelated to highly likely to be related); and its intensity (ranging from transient and without repercussions to life threatening). All these parameters will be analyzed. All possible measures will be taken by the investigation team to ensure the safety of the patients and to respect relevant laws and ethical rules. In accordance with ethical considerations (more details are provided in the “Ethical Considerations” section), an assessment of whether an adverse event can be assigned to a specific reason will be carried out for all serious adverse events.

### Ethical Considerations

The protocol received approval from the West II Ethics Committee in Angers, France (2014/30) and was approved by the French National Agency for the Safety of Medicines and Health Products (ID-RCB: 2014-A01397-40). One amendment to the protocol was submitted and approved by regulatory authorities on June 2015. The trial was conducted in compliance with French laws relating to research involving the human person. The study complied with the E6(R2) and E2B(R3) guidelines of the International Council for Harmonization of Technical Requirements for Pharmaceuticals for Human Use (ICH), which address the following topics: good clinical practices and management and electronic transmission of adverse events. The study also complied with the French Public Health Code, the French National Commission for Information Technology and Freedom, the European Union Clinical Trials Directive (2001/20/EC), and the Helsinki Declaration of 1975 and its revisions (including the Ethical Principles for Medical Research Involving Human Subjects, Tokyo 2004) and other requirements, as appropriate. A verification of the consent and emergency procedures was carried out after each inclusion, followed by an auditing of the files at regular time intervals. The study was registered on ClinicalTrials.gov (NCT02393014).

## Results

The first participant was included on March 16, 2015. We preselected 278 patients, of whom 266 enrolled in the study (low fall risk group: 75 patients, moderate fall risk group: 105 patients, and high fall risk group: 85 patients).

We will not include more patients. We have not yet analyzed the results, but our first impression is that the high fall risk group was only able to complete exercise 1 (ie, staying out of bed for at least 3 hours daily), the moderate fall risk group was able to complete exercises 1 and 2 (ie, they could also complete the 2-minute balance exercise), and the low fall risk group was able to complete all the physical activity exercises.

Despite this first impression, the program does not seem to have been perceived as difficult or time consuming by the health care team. Detailed statistical analyses will make it possible to explore in detail the results of this study and to meet its objectives.

## Discussion

The objective of this study is to verify the usability of carrying out a physical activity intervention, including 3 types of exercise, in older patients hospitalized at a geriatric acute-care unit who were at low, moderate, or high risk of falls. These exercises focused on strengthening the lower-limb muscles and improving postural balance. We hypothesized that this type of intervention would be usable, which we defined as follows: (1) 80% of the patients would be able to perform each type of daily exercise (the primary objective), and (2) the health care team would perceive the intervention as feasible in terms of its difficulty and the time required to carry it out (secondary objective B). This study also explored two other factors: (1) how the difficulty of each exercise differed for patients with different fall risk levels (ie, secondary objective A, with the intention of improving this type of program in future interventions) and (2) the potential benefits of this type of intervention (ie, secondary objective C) by comparing performance on the FTSS test before and after the intervention. To date, 266 inpatients have been enrolled. We have not analyzed the results, but our initial observations suggest that the usability of each type of exercise for the patients depends on their fall risk level, and that the program has not been perceived as difficult or time consuming by the health care team.

Recently published meta-analyses describing the role of physical activity in preventing falls by patients in posttreatment and rehabilitation wards first reported the effectiveness of exercise programs in 2012 [7], then showed less certainty in 2018 [18]. These meta-analyses also highlighted the fact that there have been very few studies conducted in geriatric acute-care units and that the data were divergent, with some studies showing efficacy and others not. The recent SPRINTT (Sarcopenia and Physical Frailty in Older People: Multi-Component Treatment Strategies) study [19] was a randomized controlled trial aimed, in part, at testing a physical activity intervention that appeared comprehensive (including aerobic endurance, gait, balance, flexibility, and resistance exercises) and monitored the intensity of the exercises to protect the frail, older participants. However, this study does not seem to have been carried out exclusively in a geriatric acute-care unit. Our study focused on the hospital context, for which, to date, there have been few studies attempting to include a significant number of patients [7,18]. If this type of intervention is feasible and “usable” from the point of view of the patients’ abilities and the perceptions of the health care team, we expect that it will reduce the risk of falling. Intervention may also reduce the frequency and severity of iatrogenic events during the hospitalization of older patients. Iatrogenic adverse events can be defined as any unintended injury or complication caused by health care management itself, rather than by the underlying disease process [20]. Sourdet et al [21] recently showed that the majority of iatrogenic events can be prevented, in part by keeping patients active and by engaging them in physical activity interventions. Martinez-Velilla et al [20] pointed out that iatrogenic disability can result from one or more iatrogenic adverse events occurring during hospitalization, including three factors: (1) the patient's pre-existing frailty, (2) the severity of the disorder leading to the admission, and (3) the hospital process of care. They also emphasized the impact of hospitalization itself, which can force the patient to be severely sedentary (ie, to stay in bed or in a chair for a long time), and the positive impact of physical activity intervention. Our physical activity program followed the recommendations of HAS [11]. It partially followed the guidelines of the International Conference on Frailty and Sarcopenia Research (ICFSR) [10]. The ICFSR guidelines focus on frailty and the prevention of sarcopenia. They recommend including aerobic exercises in addition to resistance and balance exercises. Our physical training intervention did not include aerobic exercises. Exercise 1, staying out of bed for at least 3 hours, did not engage the patients in exercise with an intensity and duration sufficient enough to be considered aerobic. Moreover, for some patients with a high fall risk level (who were usually frail), getting them out of bed for at least 3 hours, even if they only stayed seated on a chair or walked a little, was already very difficult to achieve. Another difference with the ICFSR guidelines is that our intervention did not quantify resistance or balance exercises by the number of trials, but rather by the total volume of work (2 minutes of balance and getting up from a chair 5 times). Nevertheless, the ICFSR guidelines conclude that there is currently no data that can be used to identify the optimal physical exercise required in terms of frequency, intensity, time, or type [10]. Our study may provide new information on the frequency, time, and type of physical activity interventions suited to geriatric acute-care units.

Our study presents several limitations that have already been identified. First, it was not possible to quantify the type of activities carried out during exercise 1. Managing the specific activities in this exercise would have taken too much time for the caregivers. Second, the modality of the other two exercises was not well detailed. Because of the diversity of the patients, it was necessary to let the caregivers adapt the exercises. This caused us to lose several interesting types of data (eg, the number of trials needed to reach 2 minutes or the number of times an exercise was performed on one or both feet). Third, we did not use validated scales to evaluate the perceived feasibility from the point of view of the health care team. Nevertheless, we consider that the scales we used were informative. Finally, we used only the FTSS test to assess lower-limb strength. It would have been more interesting to use the Short Physical Performance Battery [22], which includes the FTSS test.

If we verify our hypotheses on usability and if we observe a beneficial effect for the patients, we will plan a larger-scale experiment in which we will investigate the effects of this type of intervention during and after hospitalization on the risk of falling, level of frailty, level of autonomy, length of stay in the geriatric acute-care unit, recurrence of falls, and rehospitalization. Such a study would allow us to better define the frequency, intensity, time, and type of exercise for patients with different fall risk levels.

## Abbreviations

AGGIR: Autonomy Gerontology Groups Iso-Resources
BGA: Brief Geriatric Assessment
CHU: Centre Hospitalier Universitaire
COMETE: Cognition, Mobilité Et TEmporalité
CYCERON: Cyclotron-Chimie-Positron
FTSS: Five Times Sit to Stand
GIR: Groupes Iso Ressources
HAS: Haute Autorité de Santé
ICFSR: International Conference of Frailty and Sarcopenia Research
ICH: International Council for Harmonization of Technical Requirements for Pharmaceuticals for Human Use
INSERM: Institut National de la Santé et de la Recherche Médicale
LPPL: Laboratoire de Psychologie des Pays de la Loire
MMSE: Mini-Mental State Examination
SFR: Structure Fédérative de Recherche
SPRINTT: Sarcopenia and Physical Frailty in Older People: Multi-Component Treatment Strategies
UNICAEN: Université de Caen Normandie
